# Neuroinflammaging and the Immune Landscape: The Role of Autophagy and Senescence in Aging Brain

**DOI:** 10.1007/s10522-025-10199-x

**Published:** 2025-02-05

**Authors:** Rajesh Tamatta, Varsha Pai, Charu Jaiswal, Ishika Singh, Abhishek Kumar Singh

**Affiliations:** Manipal Centre for Biotherapeutics Research, Manipal Academy of Higher Education, Karnataka, Manipal 576104 India

**Keywords:** Aging, Autophagy, Microglia, Neuroinflammation, Senescence

## Abstract

Neuroinflammation is closely linked to aging, which damages the structure and function of the brain. It is caused by the intricate interactions of immune cells in the aged brain, such as the dysregulated glial cells and the dysfunctional astrocytes. Aging-associated chronic low inflammation, referred to as neuroinflammaging, shows an upregulated proinflammatory response. Autophagy and senescence play crucial roles as moderators of aging and neuroinflammatory responses. The dysregulated neuroimmune system, dystrophic glial cells, and release of proinflammatory factors alter blood-brain barrier, causing a neuroinflammatory landscape. Chronic inflammation combined with deteriorating neurons exacerbate neurological disorders and decline in cognitive function. This review highlights the neuroinflammaging and mechanism associated with immune cells interplay with central nervous system and aging, cellular senescence, and autophagy regulation in the brain's immune system under neuroinflammatory conditions. Moreover, the roles of microglia and peripheral immune cells in the neuroinflammatory process in the aging brain have also been discussed. Determining treatment targets and comprehending mechanisms that influence immune cells in the aged brain is necessary to decrease neuroinflammation.

## Introduction

Aging, a complex biological process, is associated with changes at the structural, biochemical, and molecular levels in the body. Aging is associated with modifications in the brain, for instance tissues atrophy such as grey matter neurodegeneration and white matter demyelination, alterations in neurotransmitter levels by reduction in the level of cholinergic and acetylcholine receptors, and failure to clear the aggregation and accumulation of toxic and misfolded proteins in the cellular environment (Lee & Kim 2022). Aging is implicated in chronic, low-grade inflammation phenomenon called neuroinflammaging, marking disturbed proinflammatory and anti-inflammatory homeostasis, chronically activated immune cells and impaired neuronal health. Dystrophic neurites give rise to various neurodegenerative disorders (NDDs), such as Alzheimer’s disease (AD) and Parkinson’s disease (PD), and eventually lead to a decline in cognitive function (Brito et al. 2023). With aging, the immune system deteriorates, refers as immunosenescence (A. Singh et al. 2023; Yuan et al. 2021). Along with neuroinflammaging, immunosenescence dysregulates the neuroimmune system (Di Benedetto et al. 2017). Neuroinflammation is a double-edged sword in central nervous system (CNS); it is believed to act as a neuroprotectant by activating glial cells mediated innate immune response, aiding neuronal repair by releasing BDNF; however, chronic neuroinflammation accelerates neurodegeneration by toxics accumulation such as ROS (Fuster-Matanzo et al. 2013; Kempuraj et al. 2017). Revealed by earlier studies, the prominent characteristics of brain aging include 1. dysfunctional mitochondria, 2. oxidative damage, 3. dysregulation of energy metabolism, and 4. impaired autophagy. 5. compromised DNA repair, 6. dysregulated neurotransmission, and 7. inflammation (Mattson and Arumugam 2018).

The CNS has been an immunologically privileged site for a long time, as grafts were not rejected when implanted in the brain compared with other organs. This property is attributed to the selectively permeable blood–brain barrier (BBB), allowing only specific and small molecules to enter the brain from the blood, and the absence of typical lymphatic vessels prevents pathogen entry and protects from inflammation. Brain microglia respond to injury and modulates immune response without adverse effects. CNS actively interacts with peripheral immune cells through cytokines and neuropeptides. This complex interaction maintains immune privilege in CNS (Bauer et al. 2024).

Senescence is a persistent cell cycle arrest associated with the expression of p16^INK4a^ and p21^Waf1/Cip1^. Nelke et al. (2022) reported that senescence occurs in neuroinflammatory disorders and might play a crucial role in neuronal disorders (Nelke et al. 2022). In addition, cells that are undergoing senescence exhibit a senescence-associated secretory phenotype (SASP) that produces various proinflammatory molecules. Thus, the accumulated senescent cells induce localized inflammation in neighboring tissues. Hence, clearing senescent cells or inhibiting their aggregation results in decreased neuroinflammation and improved cognition (Matsudaira et al. 2023).

From the finding, microglia, brain immune cells formed from precursors of embryonic sacs, self-renewal properties, considered a distinct cell population in the CNS in aged brain, released elevated inflammatory molecules, impairing synaptic function and neuronal structures and cognitive decline (Paolicelli et al., 2022; Füger et al. 2017).

The activation of autophagy can achieve the clearing of senescent cells, remove damaged cargo via lysosome-dependent degradation, and also clear damaged mitochondria for the normal regulation of cell function and metabolism (Chou et al. 2023a, 2023b). Moreover, impaired autophagy leads to increased SA-β-gal activity in senescent neurons, suggesting that dysfunction of the autophagy process leads to neuronal senescence (Moreno-Blas et al. 2019a, 2019b).

**Fig. 1:**
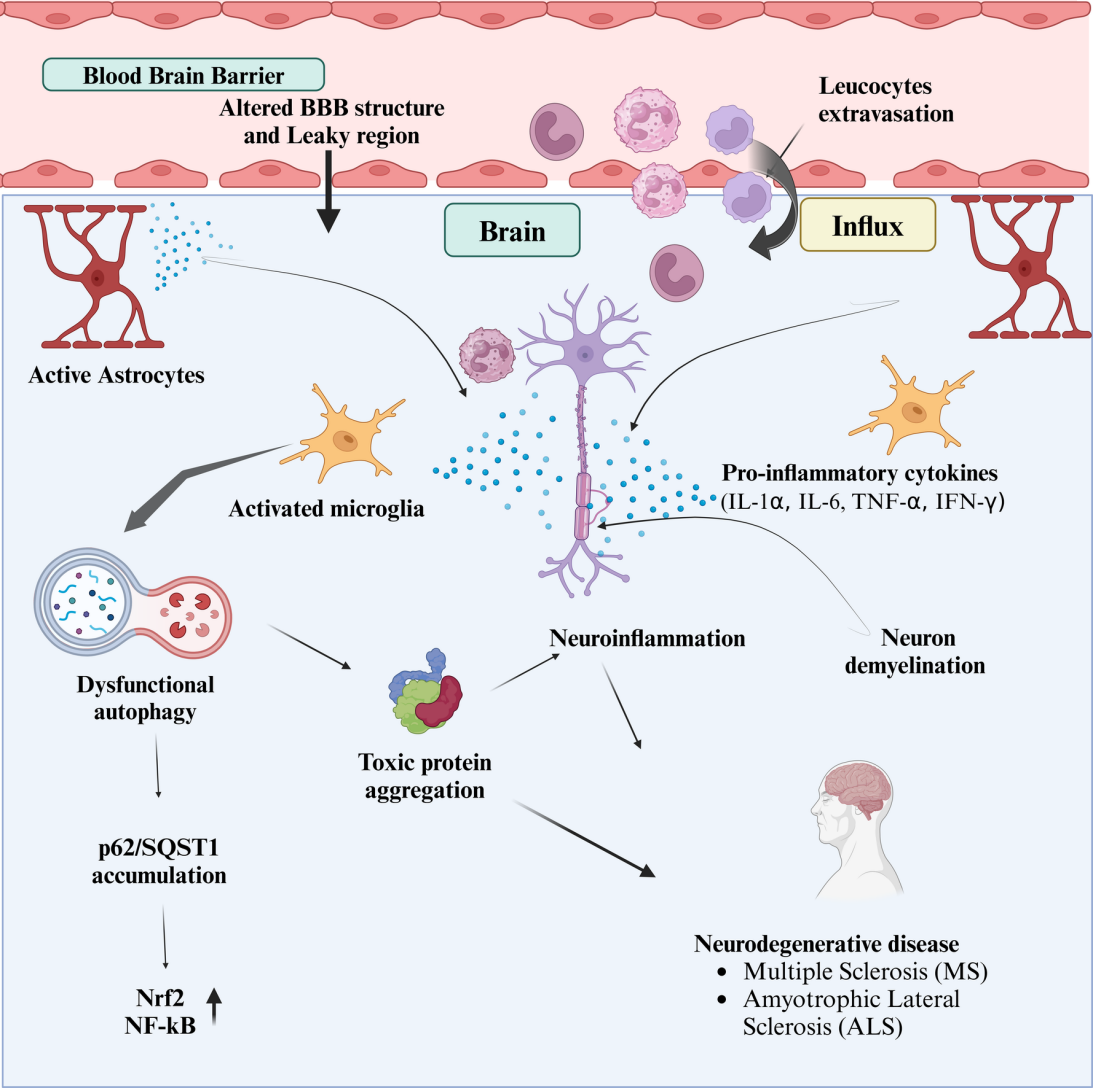
Compromised tight junction structures in the blood–brain barrier cause peripheral immune cell infiltration and leucocyte extravasation, resulting in inflammation. Activated astrocytes and reactivate microglia release proinflammatory cytokines that promote immune cell aggregation and contribute to neuronal demyelination, ultimately causing neuroinflammation. Additionally, the aggregation of toxic proteins exacerbates inflammation. Accumulate p62 in dysfunctional autophagy leading to upregulated Nrf2 production and NF-kB expression

Evidence has shown the close interaction between senescence, autophagy, and neuroinflammatory responses, which emphasizes their roles in brain aging. In contrast, autophagy clears the accumulated toxic and damaged cellular components and rescues the cells that are undergoing senescence; thus, a decrease in SASP decreases neuroinflammation. Conversely, impairment of the autophagy process exacerbates cellular senescence and neuroinflammatory responses, creating a vicious cycle that increases brain aging known as neuroinflammaging (Shafqat et al. 2023). This review focuses on providing a clear understanding of the interconnected mechanisms of autophagy, senescence, and neuroinflammation, thus highlighting critical signaling pathways that integrate and regulate brain aging and neurodegenerative diseases (Fig. 1).

## Autophagy in inflammaging and longevity

### Role of autophagy in inflammaging

Autophagy, a cellular recycling process, clears aggregated toxics protein and maintains cellular homeostasis. As organisms age, impaired autophagy, senescent neuronal and glial cells, immune response dysregulation, and chronic inflammation lead to cellular homeostasis perturbation and exacerbate toxic aggregation and neuroinflammation. Exacerbated neuroinflammation and neuroinflammaging hinder neurological health and fosters neurodegeneration and cognitive decline. Neurons, peripheral immune cells, and glial cells are closely associated with each other and are essential for regulating cellular immune responses and homeostasis (Füger et al. 2017). As brain-residing immune cells, microglia preserve homeostasis and function in the immune surveillance of foreign pathogens for detection and elimination (Latham et al. 2023; Sil et al. 2023).

In response to stimulus, microglia either adapts to the state dominated by inflammatory mediators or to the state characterized by anti-inflammatory mediators for maintaining homeostasis. In response to injury and inflammation, the brain produces neuroprotective microglia, promoting neurogenesis, lowering inflammation, and inducing phagocytosis for debris removal (Z. Chen and Trapp 2016). Reactive microglial populations surrounding toxic aggregates are also known as disease-associated microglia (DAMs) (Latham et al. 2023). Neurotoxic phenotype activation occurs upon the binding of PAMPs from bacterial fungi and DAMPs from damaged organelles, such as nucleic acids, to pattern recognition receptors (PRRs) and toll-like receptors (TLRs) (Latham et al. 2023). Two major pathways are responsible for inflammatory signaling in the neurotoxic phenotype, namely, the NF-κB and MAPK pathways (Doens and Fernández 2014; Latham et al. 2023). The expression of MHC complexes MHCI and MHCII, particularly in the hippocampus, is upregulated during aging (Kellogg et al. 2023; VanGuilder et al. 2011). Upregulated MHC complexes are associated with increased toll-like receptor expression and dysregulated autophagy (Kellogg et al. 2023; VanGuilder et al. 2011). These findings indicate that microglia are crucial for autophagy and senescence regulation and that reactive microglia secrete chemokines such as CCL3, CCL4, and CCL5, activate neuronal CCR5 receptors, activate the mTORC1 pathway, impair autophagy and disrupt protein clearance in HD and PS19 tauopathy model mice. In contrast, the depletion of CCR5 receptors has a neuroprotective effect (Choi et al. 2023; Festa et al. 2023). Dysregulated microglia and neuroinflammation cause impaired autophagy (Sil et al. 2023). Reports have suggested that the activation of autophagy in microglia, especially in proximity to amyloid plaques, in experimental AD mice, reduces AD pathogenesis. Microglial defective autophagy in AD mice causes microglial detachment from plaques, decreasing the number of disease-associated microglia and increasing neuropathology (Choi et al. 2023; Suelves et al. 2023). Research has revealed that class IIA histone deacetylases (HDACs), which are highly expressed in the brain, are important immune response regulators. Partitioning defective 1 (Par1), another name for microtubule affinity regulating kinase (MARK), serves as an active regulator of HDACs. Studies conducted on a controlled cortical impact (CCI) injury mouse model revealed that mice with defective Par-1b/MARK2 sustained TBI (traumatic brain injury) and exhibited an increased inflammatory response (Patil 2020). Studies have shown that the activated AMPK pathway in microglia makes microglial dynamic shifts from pro-inflammatory secretions to anti-inflammatory mediators by blocking the inflammatory NF-κB pathway (Patil 2020; Saito et al. 2019; Velagapudi et al. 2017; Xiang et al. 2019). Astrocytes influence microglia by cytokines secretion, namely TNF-α, and IL-1β, during stress. Reactive astrocytes play a dual role in repair by debris clearance or exert neurotoxicity through dysregulated glutamate transporter, high NO production, and dysregulated AQP-4, causing cytotoxic edema (Yu et al. 2021).

### Role of autophagy in longevity

Autophagy is essential for promoting healthy aging and increasing lifespan by regulating cellular homeostasis. From the study, hormetic factors induce autophagy influencing longevity by removing damaged cellular components and debris that accumulate with age, thus maintaining cellular function and decreasing oxidative stress. Mitochondria is an energy ATP source and ROS producer. Acetyl-L-Carnitine (ALC) supplements enhance mitochondrial biogenesis, regulate oxidative stress, and promote longevity. ALC improves cellular resilience against aging and neurological disorders by modulating Nrf2 expression, including HO-1 and HSP upregulation (Calabrese et al. 2010). In mammals, dietary interventions such as calorie restriction and intermittent fasting have been shown to prolong lifespan by stimulating autophagy mechanisms (A. Singh et al. 2023; Yuan et al. 2021). Early studies by Mark Mattson’s showed that CR may contribute to enhance the lifespan. Furthermore, IF has become a popular approach compared to traditional CR. It involves complete fasting for a period of more than 12 h to enhance the physiological benefits. Current research have broadened understanding by exploring the alternative strategies that may increase longevity and health span without any strict dietary intervention (Sharma and Kaur 2021). Deprivation of nutrient uptake, IF, CR, oxidative stress, and hypoxia conditions that can produce moderate physiological stress that will result in hormesis (Moore 2020). Thus, dietary intervention may modulate the aging process. Malnourished conditions such as vitamins and nutrient deficiencies hinder cognitive function and impair neurotransmitter synthesis (Abdel-Rahman et al. 2024). The study found that protein deficiency severely impacts hypothalamic serotonin and dopamine levels. Deficiency of micronutrients such as vitamin B12 impacts neurotransmitter synthesis along with neuronal myelination (Sethi et al. 2022; Venkatramanan et al. 2016). Study conducted in *C. elegans,* mild hormetic heat shock at 20ºC upregulates selective autophagy receptor SQST-1/p62 transcripts level. Enhanced SQST-1/p62 transcripts elevate autophagy and extend longevity (Kumsta et al. 2019). According to the in vitro research conducted on senescent human skin fibroblast, using immunofluorescence, five-fold increases in basal LC3 was found, and no difference in thick skin biopsies and photo-exposed and photoprotected region was observed (Demirovic et al. 2015).

Pathways such as the AMPK and mTOR pathways impact the molecular basis of the impact of autophagy on lifespan. A decrease in the mTOR pathway and inflammatory process, an inhibitor of autophagy, has been shown to increase lifespan across various species (Li and Chen 2019). Various autophagy activators, such as metformin, Torin 1, and rapamycin, can restrict the activity of mTOR; thus, they have the potential to increase autophagy and delay aging. The alleviation of proinflammatory molecules associated with senescence through autophagy can contribute to longevity and tissue health.

In these studies, intermittent fasting and calorie restriction increased autophagic activities. The pharmacological agent spermidine has been shown to activate autophagy in an experimental model (Hofer et al. 2024; Ni & Liu 2021). Spermidine, as an endogenous substance, attracts as therapeutics without adverse effects and tolerability. Spermidine clears damaged cells via MAP1S-mediated autophagy also reduces oxidative stress, and metabolic restoration achieved by this intervention in MAP1S^+/+^ and MAP1S^−/−^ mice (Yue et al. 2017). Spermidine oral administration in APP-PS1 shows a reduction in Aβ plaques and exerts anti-inflammatory inducing microglia transcriptomics alteration, and actively regulates TREM2 receptors (Freitag et al. 2022). Researchers can mitigate both aging and several diseases via the use of autophagy-based therapies.

## Cellular senescence in brain aging

Cellular senescence is a state in which cells undergo permanent cell cycle arrest, which is associated with several macromolecular alterations as well as hypersecretion. Senescent cells naturally get accumulated  in the brain as people age thus, it leads to age-related disorders and impaired neurogenesis (Melo dos Santos et al. 2024). The development of neurodegenerative processes and the development of cellular damage are intimately linked to aging in the brain in terms of central nervous system health. Cellular senescence, an irreversible growth halt with an excessive and proinflammatory phenotype known as SASP, is a major factor in the deterioration of the brain's structure and function (Gorgoulis et al. 2019). Upregulated expression of cyclin-dependent kinases (CDKs), specifically CDKN2A/p16^Ink4a^ and CDKN1A/p21^CIP1/WAF1^, is the primary cause of cellular senescence, a basic biological process (Idda et al. 2020). Significant alterations in cellular morphology and architecture accompany the cessation of this growth. Senescence-associated β galactosidase (SA-β-gal) activity is a major indicator of enhanced lysosomal activity in senescent cells and typically increases the cell volume (Melo dos Santos et al. 2024).

A decrease in the nuclear lamina protein Lamin B1 (LMNB1) and persistent nuclear presence of response proteins from DNA damage, such as γ-H2AX, are two indicators of nuclear changes that contribute significantly to cellular senescence (Rodier et al. 2011). These modifications are predominantly caused by the SASP. Bioactive lipids, proinflammatory cytokines, chemokines, microRNAs, extracellular matrix proteases, and extracellular vesicles are all part of the vast array of substances that constitute the SASP. These bioactive substances control a variety of biological processes via both paracrine and autocrine signaling (Basisty et al. 2020).

During neuronal aging, cell senescence increases, which causes SASP factors to be released over time. This creates an environment that favors dysfunction and illness and adds to inflammaging, a chronic inflammatory condition linked to aging (Franceschi et al. 2018). Notably, therapies that target senescent cells have shown potential for reducing dysfunction and prolonging life expectancy (Chaib et al. 2022). In the aging brain, various cell types exhibit senescence, each with distinct characteristics and roles.

### Senescence in microglial and neuronal cells

While neurons do not exhibit replicative senescence features such as telomere shortening, aging neurons display several hallmarks of senescence. These include dysfunction of the mitochondria, damage to DNA, SASP, nuclear morphological changes, macromolecule aggregate accumulation, elevated levels of β-gal, and increased cell cycle inhibitor expression (Jurk et al. 2012). During aging, microglia exhibit a deformed structure, abnormal functioning and increasing vulnerability to senescence and inflammation (Sil et al. 2023).

DNA damage is a well-established driver of senescence in cells undergoing mitosis, primarily resulting from errors in replication and stressors such as oxidative stress and oncogenesis (J.-H. Chen et al. 2007). Aged neurons also accumulate markers of DNA damage. Single-neuron sequencing of the hippocampus and prefrontal cortex of individuals aged 4 months to 82 years revealed a near-linear increase in somatic single-nucleotide variants with age (Lodato et al. 2018). Senescence is intimately associated with damaged DNA, predominantly double-strand breaks (Sedelnikova et al. 2004). In older mice, Purkinje and cortical neurons contain the γ-H2AX marker, which is suggestive of DSBs (Moreno-Blas et al. 2019a, 2019b). Oxidative stress prominently damages DNA, including single-strand breaks (SSBs) and base alterations. Hippocampal cerebellar granule neurons, pyramidal neurons, and granule neurons in aged mouse brains exhibit elevated nuclear DNA SSBs, which frequently accumulate at genomic enhancers (Wu et al. 2021). Furthermore, aged rat neurons develop 8-oxoguanine (8-oxoG) lesions, whereas aged human neurons exhibit significant damage to mitochondrial DNA (Bender et al. 2006). This vulnerability is likely due to the high oxidative phosphorylation demands of neurons and exposure to elevated free radical levels (Chou et al. 2023a, 2023b).

#### Hallmarks of neuronal senescence

Key drivers of neuronal senescence include DNA damage, mitochondrial dysfunction, SASP, and increased levels of cell cycle inhibitors. Other features, such as lipofuscin accumulation, changes in nuclear morphology, and increased SA-β-gal activity, are often observed in neurons from aged animals and extended cultures but are not essential for senescence development (Moreno-García et al. 2018).

Morphological changes, such as changes in shape and size and decreased lamin-B1 expression, a biomarker of senescence, are observed in aged neurons (Matias et al. 2022). Although neurons naturally express low lamin-B1 levels, nuclear envelope abnormalities have been observed in the cortical neurons of old rat brains and extended cultured neurons, elevated lamin-B1 in aged neurons despair shape, affecting neuronal health and function (Gorostieta-Salas et al., 2021; Ishikawa & Ishikawa 2020).

Lipofuscin, a yellow–brown auto-fluorescent pigment comprising lipids, metals, and misfolded proteins, progressively accumulates in the lysosomes of postmitotic cells, including aged neurons and glia (Moreno-García et al. 2018). This pigment is frequently found in aged cortical and Purkinje’s neurons and is correlated neurons structure deformities, alter cytoskeleton morphology and dysregulate autophagic processes (Moreno-García et al. 2018).

#### Cellular senescence in glial cells

Cellular senescence, an inflammatory secretory condition that a fraction of cells display is associated with aging in mammalian species and tissues. While acutely beneficial for wound healing and tumor suppression, chronic senescence is detrimental (Reyes et al. 2022). Senescent glial cells in chronic neuroinflammation implicate neurological disorders. This senescent glial cell accumulation is believed to cause neuroinflammation and neuronal injury (Martínez-Cué & Rueda 2020). From the research, accumulated senescent glial cells affect α-synuclein clearance and exacerbate PD pathology (Hong et al. 2024). Current insights are largely derived from animal models or in vitro studies where senescence is artificially induced by oncogenic Ras expression, DNA damage by ultraviolet exposure, and telomere shortening in telomerase-deficient mice (Sharpless and Sherr 2015). Studies in aging mice have shown that periodically clearing senescent cells can extend lifespan, improve tissue health, and alleviate age-related diseases (Baker et al. 2011). These improvements are attributed primarily to the reduction in the SASP and the associated inflammatory environment. However, key questions about nature and the impact of senescent cells remain unanswered. It is uncertain whether these models accurately reflect natural senescence, as identifying and manipulating naturally occurring senescent cells in vivo remains challenging. Consequently, the mechanisms and implications of senescence in living organisms, particularly their role in tissue aging, remain poorly understood.

AP1 activation occurs in a subset of glial cells that exhibit traits and biomarkers like those of senescent mammalian cells in aging fly brains, according to a study that revealed naturally occurring senescent glia in the aging Drosophila brain shows lipid droplet accumulation in non-senescent glia is associated with these AP1^+^ glia and results in the development of a neuronal mitochondrial failure response. Similarly, in vitro research has indicated that AP1 activity in human senescent fibroblasts facilitates LD development in non-senescent fibroblasts. Targeting AP1 activities in senescent glial cells can extend longevity in Drosophila brain (Byrns et al. 2024). Additionally, senescent astrocytes cause oligodendrocytes to undergo senescence affecting myelination, indicated by increased levels of p21 and senescence-associated β-galactosidase (SA-β-gal). These compounds also increase glutamate release. Neighboring microglia are negatively impacted by the SASP released by senescent astrocytes (Y. Wang et al. 2024; Yu et al. 2021). Neurodegenerative disorders are exacerbated by senescent, microglia that release SASP components; exhibit SA-β-gal overexpression, p16, and p21; and promote Aβ. Overall, aging in the brain may be caused by the interaction of neurons and senescent glial cells (Hong et al. 2024).

## Senescence-associated secretory phenotypes (SASPs)

Neurodegenerative processes in the brain are facilitated by cellular senescence. Senescent cells are relevant to aging-related neuroinflammation. The SASP is a marker of senescence that may appear in both neurons and glial cells. SASP secretion involves chemokines, proteases, cytokines, and lipids. IL-6 is a major SASP component that is secreted by aged neurons that has been cultured for a long period. The secreted SASP has been shown to stimulate astrocyte proliferation and senescence induction in the fibroblasts of mouse embryos (Moreno-Blas et al. 2019a, 2019b), highlighting its role in paracrine senescence and chronic inflammation (Acosta et al. 2013). Reactive astrocytes, for example, contribute to neuronal aging and inflammation in the brain by releasing proinflammatory cytokines, activating complement systems, and secreting neurotoxins (Yu et al. 2021). In the cortical neurons of aged rat brains, the transcription factor GATA4, a crucial SASP regulator, builds up and stimulates the expression of factors such as monocyte chemotactic protein-1 (MCP-1). Microglia, astrocytes, and endothelial cells can all experience senescence because of this paracrine signaling. By losing their supporting functions and developing proinflammatory and neurotoxic phenotypes, aged glial cells worsen neuronal senescence (Salas et al. 2020).

## Senescence-associated secretory proteins and their role in neurodegeneration

**Fig. 2:**
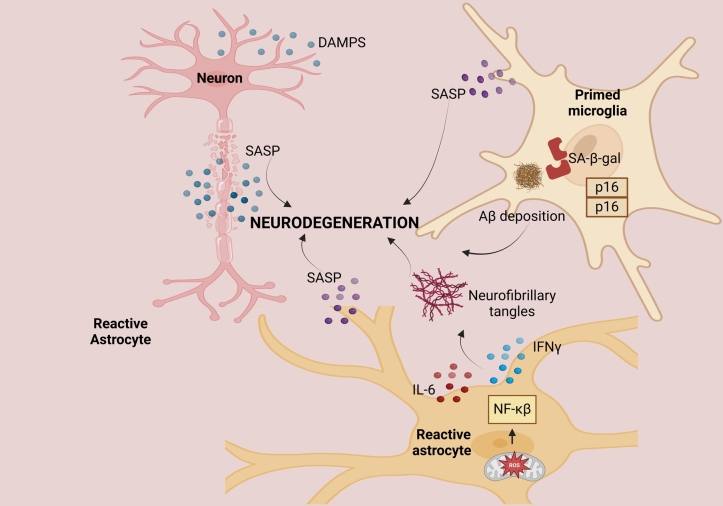
Senescent glia and neurons promote neuroinflammation and neurodegeneration via SASP secretion, DAMP release, and mitochondrial dysfunction. Senescent astrocytes drive Aβ accumulation, tau hyperphosphorylation, and oligodendrocyte senescence while impairing microglia. Senescent microglia secrete SASP components and exacerbate Aβ pathology

Senescent cells influence the microenvironment by releasing a broad range of chemicals, including growth factors, proteases, cytokines, extracellular matrix components, and nonprotein secretions (Sikora et al. 2021). Senescent cells are relevant to neuroinflammation related to aging. The SASP is a marker of senescence that may appear in both neurons and glial cells. This phase releases bioactive substances, such as cytokines, chemokines, and enzymes, that degrade the extracellular matrix, which further enhances the proinflammatory environment in the aging brain. Moreover, senescent cells produce biological factors known as danger-associated molecular patterns (DAMPs) from neighboring cells, promoting the expression of inflammatory genes in brain cells (Chinta et al. 2015; Wissler Gerdes et al. 2020) (Fig. 2).

**Fig. 3:**
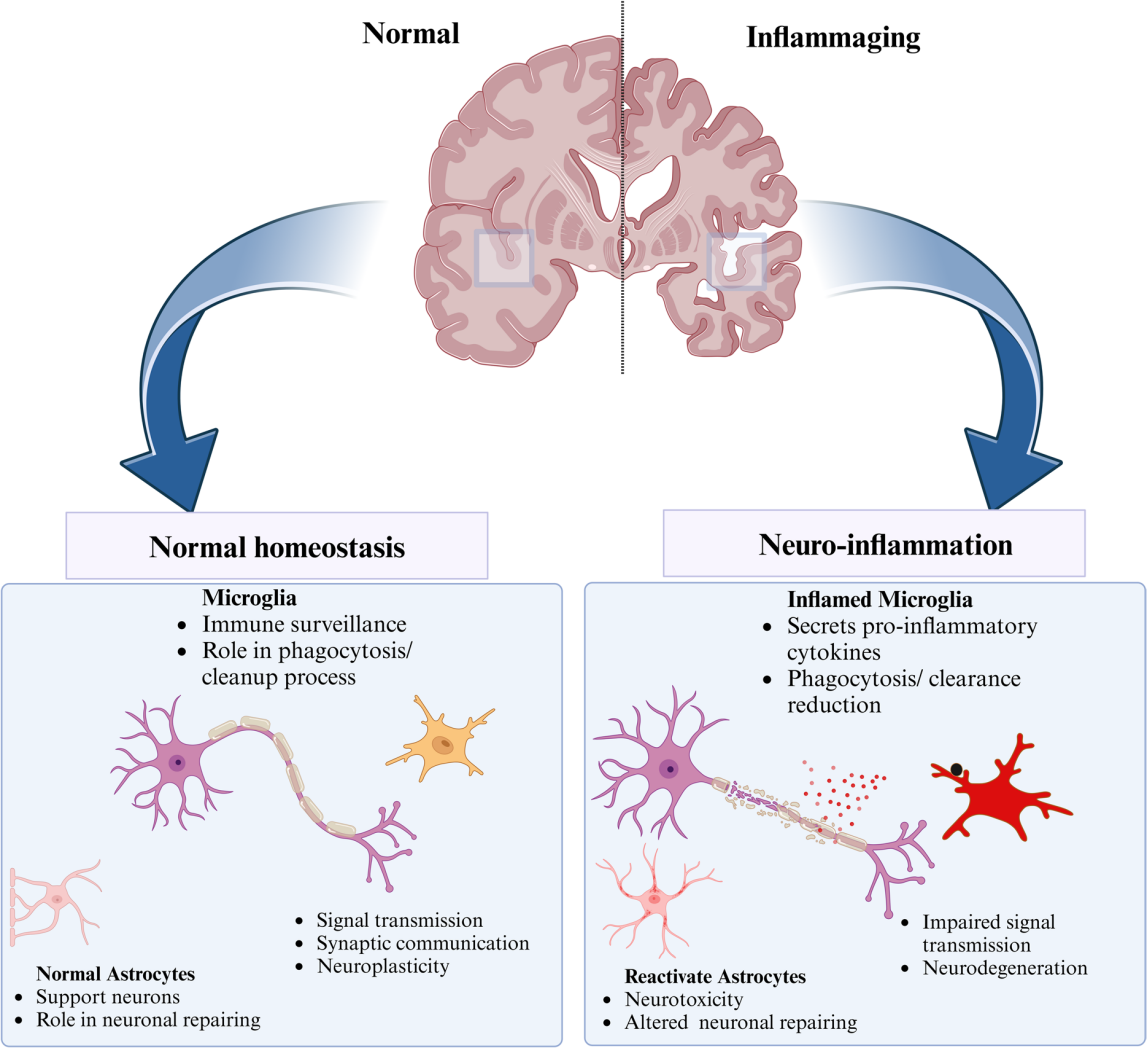
Comparison of normal and inflamed brains. In normal homeostasis, microglia perform a cleanup process, and astrocytes support the repair process and neuronal function. In neuroinflammation, inflamed microglia release proinflammatory cytokines and impair the cleanup process. Activated astrocytes disrupt neuronal repair, promoting neurotoxicity

Senescent astrocytes produce more ROS because of mitochondrial malfunction. The NF-κB pathway is triggered, leading to the release of IL-6 and IFNγ. These substances cause beta-amyloid buildup and tau hyperphosphorylation, which in turn results in the formation of neurofibrillary tangles pathology (S. Singh and Bhatt 2024) (Fig. 3). Senescent cells also overexpress and produce IL-1α and IL-1β, which affect nearby cells by activating the AP-1 and NF-κB signaling pathways via the IL-1 receptor/Toll-like receptor family (Haga and Okada 2022).

Senescent cells release significant quantities of insulin-like growth factor-binding proteins (IGFBPs), colony-stimulating factors (CSFs), osteoprotegerin, prostaglandin E2 (PGE2), matrix metalloproteinases (MMPs), NOD-like receptor pyrin-domain containing 3 (NLRP3), LC3II, neurofibrillary tangles (NFTs), nitric oxide (NO) and reactive oxygen species (ROS), and regulators, altering the IGF/IGF receptor network.

Colony-stimulating factors (CSFs), osteoprotegerin, prostaglandin E2 (PGE2), and the enzyme that synthesizes PGE2, and Cox-2 are secreted by senescent cells, which promote inflammatory reactions and aging-related alterations (Z. Han et al. 2024). Matrix metalloproteinases (MMPs), such as MMP-1, MMP-3, and MMP-10, help rebuild the extracellular matrix and regulate the activity of SASP cytokines. To influence cellular connections, they can cleave chemokines such as MCP-1 and IL-8 (Coppé et al. 2010) (Fig. 2).

In vitro studies have shown that the protein IGFBP3 induces cell senescent by proliferation inhibition through telomeres shortening and enhanced SA-β-gal (Kwon et al. 2023). Senescent cells increase proteolytic activity and strengthen growth arrest by inhibiting tissue plasminogen activators (tPAs) and their inhibitors (PAI-1/2). Aged cells have markedly higher levels of these PAI-1/2. From in vitro studies, SASPs, along PA-1/2 secretion from senescent cells, hinder t-PA activity from degrading IGFBP3 (Elzi et al. 2012; Eren et al. 2014).

Senescent cells release NO and ROS because of altered metabolic activity. These substances affect the differentiation of neighboring cells, encourage the growth of cancer, and cause tissue deterioration and aging. Together, these secretions alter the tissue microenvironment, promoting inflammatory responses, the growth of tumors, and age-related deterioration (Chou et al. 2023a, 2023b)**.**

## Cellular senescence and its association with Alzheimer’s disease

Aging is recognized as the prominent factor in AD progression, although the mechanisms linking aging to increased susceptibility are still unclear. AD is an NDD that affects more than 30 million people worldwide and has no effective treatments due to incomplete knowledge of its etiology and pathogenesis. AD affects nearly half of the people aged 85 and older, and its risk doubles approximately every five years after the age of 65.

Cellular senescence is a major contributor to the development of many aging-related diseases, including AD (Baker and Petersen 2018; X. Han et al. 2020), according to the findings of human and animal studies (Gerenu et al. 2017; Tacutu et al. 2011). Two of the neuropathological hallmarks of AD are neurofibrillary tangles (NFTs), which are formed by intracellular aggregates of hyperphosphorylated tau proteins (Montine et al. 2012). Although the precise roles of tau and Aβ in neurodegeneration and memory loss are still under debate, there is mounting evidence that both Aβ and tau pathologies are powerful inducers of cellular senescence.

Senescent cells have been found in AD mouse models that overexpress tau or Aβ proteins (P. Zhang et al. 2019) as well as in the brains of AD patients (X. Han et al. 2020). Research has shown that pharmacologically or genetically eliminating senescent cells improves memory in these animal models while lowering tau pathology and Aβ buildup in the brain (P. Zhang et al. 2019). These results imply that cellular senescence contributes to the neuropathological processes induced by tau and Aβ and may worsen these disorders.

The development of prevention and treatment methods for AD requires an understanding of the process underlying brain cells senescence during aging and AD, as well as how senescent cells contribute to neurodegeneration.

## Role of autophagy and senescence in brain immune cells

**Fig. 4:**
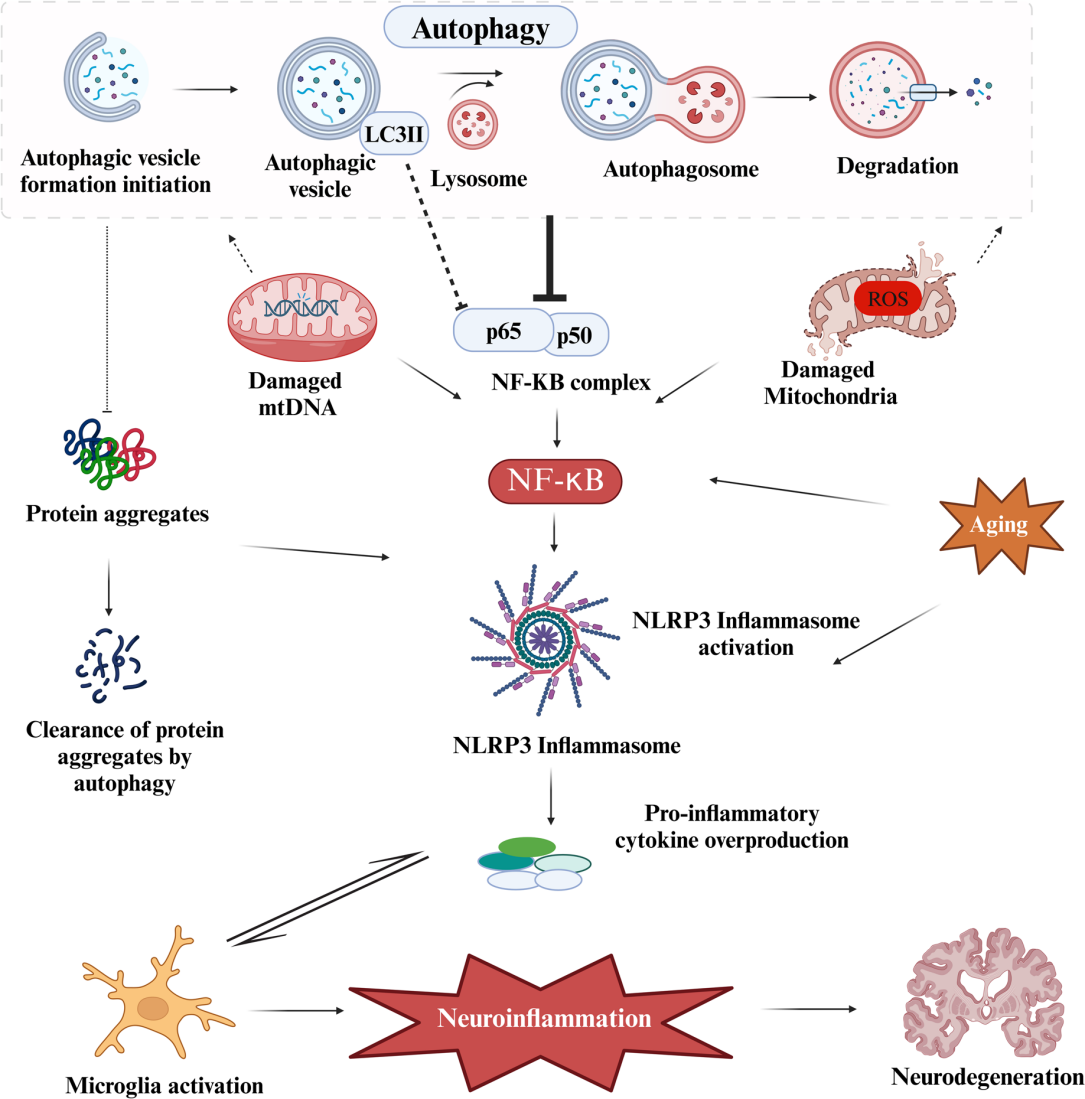
Crosstalk between NF-κB and the autophagy pathway. Autophagy degrades damaged mitochondria and clears protein aggregates, thereby inhibiting NF-κB signaling. However, dysfunctional autophagy causes the activation of the inflammatory NLRP3 pathway. Reactive microglia and inflammatory pathways release proinflammatory cytokines, which promote neuroinflammation and, consequently, neurodegeneration disorders

Crosstalk among autophagy, senescence, and brain immune cells is critical for maintaining brain health, maintaining cellular homeostasis, and balancing brain immune cells and neuronal health. Although they share few similarities and are interconnected with the development of neurodegenerative disorders, they share common features. Both protect cells from external toxicity, such as radiation, internal stress, telomere shortening, DNA damage, and impaired mitochondria. Autophagy is crucial for senescence inhibition or anti-senescence (Cassidy and Narita 2022; Young et al. 2009). Microglia and astrocytes, central nervous system (CNS) immune cells, are used for the cellular autophagy process to eliminate plaques and prevent neurons and glial cells from disease (Yang et al. 2010). As cell age and stress molecules hinder the cellular autophagy process, impairment of autophagy drives microglia into senescence and hinders cell multiplication, a reduction in autophagic flux leads to aggregation of damaged cellular components, particularly in neuronal and microglia, causing cessation of cellular proliferation, progression into the senescence stage, and the secretion of inflammatory molecules such as SASP (Suelves et al. 2023). Impaired autophagy in senescent cells causes the overproduction of inflammatory cytokines, ROS, and neurotoxic factors. In the brain immune system, SASP induces proinflammatory inflammation and neuronal tissue damage in neurogenerative disorders. Reactive microglial responses to the SASP accelerate the neuroinflammatory cascade and disrupt cognitive function and neuronal networks. Similarly, astrocytes in the blood–brain barrier are more reactive and induce an inflammatory cascade. A recent study conducted on primary microglia from mice revealed that microglia at homeostatic effectively engage with plaques and their removal, whereas senescent microglia fail to engage with amyloid plaques, consequently leading to increased neurotoxicity (Choi et al. 2023). SASP components, such as IL-1β and TNF-α, activate the inflammatory NF-κB pathway, creating a feedback loop between neuro-immunity and inflammation (Barnabei et al. 2021; Liu et al. 2017). According to previous findings, autophagy attenuates NF-κB, and the autophagy protein LC3II selectively targets the NF-kB complex and the NLRP3 pathway by degrading inflammatory molecules and preventing an immune response (Yi et al. 2019). In the central nervous system, abnormal autophagy causes overproduction of proinflammatory, hyperactivating NOD-like receptor pyrin domain containing 3 (NLRP3), exacerbating neuroinflammation, neuronal loss, neural damage, cognitive decline and diminishing neuroprotective functions (Blevins et al. 2022). Dysregulation of the interplay between autophagy and the neuroimmune system is the root cause of several NDDs, such as Huntington's disease, AD, and PD (Fig. 4).

## Immune pathways and their effects on the blood–brain barrier (BBB)

The blood–brain barrier (BBB) is the monocellular endothelial cell layer of brain capillaries, which consists of tight junctions that form a barrier that distinguishes the peripheral system from the central nervous system. The barrier allows the passage of only selected molecules, such as nutrients, and restricts the entry of harmful pathogens into the brain. The structural integrity of the BBB prevents unwanted immune responses and inflammation. Numerous factors damage the structural integrity of the BBB. Alterations in the structure of the BBB have various outcomes, including neurological diseases and neuroinflammation. During infections and diseases, immune cells release TNF-α, IL-1β, and proinflammatory cytokines, which alter the structure of the BBB tight junction structure and allow for cell infiltration and permeability (Banks and Erickson 2010; Mapunda et al. 2022). In compromised BBB structures, infiltration of peripheral immune cells, such as macrophages and T cells, leads to inflammation. During infection, lipopolysaccharide (LPS) from bacterial cells also alters the BBB structure, increasing immune cell permeation into brain tissue. Consequently, this inflammation is associated with various diseases, one of which is multiple sclerosis (MS). The mechanism underlying the entry of peripheral immune cells involves the expression of E-selectin adhesion molecules on the surface of activated endothelial cells during the inflammatory response, allowing immune cells to adhere to and extravasate leukocytes. Immune cells from the perivascular space migrate into the brain, changing the matrix components (Galea 2021).

### Role of cytokines in neuroinflammation

Cytokines, signaling molecules released by immune cells that enable cell-to-cell communication, play major roles in neuroinflammation and influence both immune cells and neurons in the brain. Two broadly categorized cytokine classes, i.e., the proinflammatory and anti-inflammatory cytokine classes. The pro-inflammatory cytokines IL-1β, TNF-α, IL-6, and IFN-γ are crucial neuroinflammatory responses, and their dysregulation exacerbates neurodegenerative disorders. IL-1β is crucial for initiating the immune response in the brain; triggering reactive microglial for overproduction of proinflammatory factors, contributing to neuronal loss (Becher et al. 2017; W.-Y. Wang et al. 2015). TNF-α promotes apoptosis, exacerbating neuroinflammation by enhancing the inflammatory response (Becher et al. 2017). IFN-γ is secreted by T cells, eliciting microglia for propagating an inflammatory cascade (Becher et al. 2017; Rhie et al. 2020). These persistent proinflammatory cytokines can cause neuronal injury and a decline in cognitive function. IL-10 and TGF-β, which are anti-inflammatory cytokines, counteract proinflammatory cytokines. These anti-inflammatory cytokines have immunosuppressive properties and regulate the immune response by limiting the production of pro-inflammatory signals (Becher et al. 2017; Y. Chen et al. 2024).

### Crosstalk between NF-κB and the autophagy pathway

The interaction between NF-κB and the autophagy pathway is essential for understanding inflammation and cellular homeostasis. Both processes regulate various biological processes, and dysregulation is associated with significant implications in disease processes. Autophagy selectively targets NF-κB pathway components and suppresses the inflammatory pathway cascade. In recent studies, the autophagic marker LC3 was shown to bind to NF-κB p65 subunits, ubiquitinate them, and degrade ubiquitinated subunits, subsequently dampening inflammatory pathways. According to these findings, the selective autophagy receptor p62/SQSTM1 targets ubiquitinated proteins required for NF-κB pathway activation, thereby hindering pathways (Kumar et al. 2022). In contrast, the NF-κB pathway plays a dual role, either by inhibiting or promoting autophagy. Under certain circumstances, NF-κB promotes autophagy by increasing the expression of the autophagy regulatory gene Beclin-1, which promotes cell survival under stress (Salminen et al. 2012).

This interaction is highly context-specific; NF-κB either stimulates or inhibits autophagy pathways during inflammation. The pro-inflammatory molecule TNFα simultaneously activates both autophagy and NF-κB, resulting in a coordinated inflammatory response. In NDDs, impaired autophagy results in persistent NF-κB activation, leading to chronic inflammation (Y. Chen et al. 2024). In cancer, autophagy inhibits NF-κB by modulating antiapoptotic signals and protects cancer cells from inflammatory pathways (Trocoli and Djavaheri-Mergny 2011).

### Interplay between AMPK and the NF-kB pathway in neuroinflammation

**Fig. 5:**
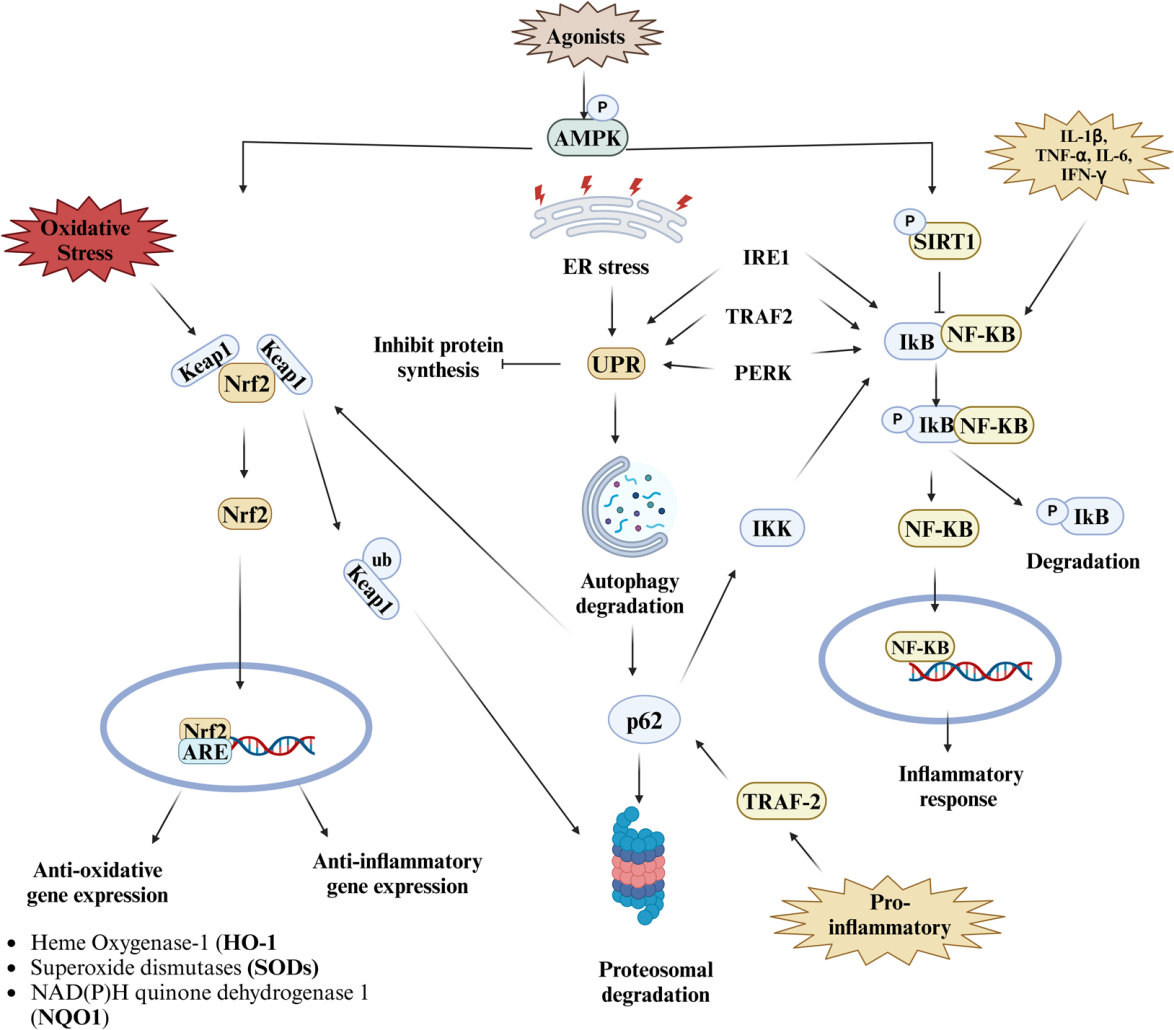
Interplay among AMPK, NF-κB, Nrf2/p62, and UPR pathways. Activated AMPK phosphorylates the downstream molecule SIRT1 and activates the Nrf2 pathway, and activated Nrf2 dissociates from the inhibitor keap1 and migrates into the nucleus, expressing anti-inflammatory genes and inhibits inflammatory signals. Due to misfolded protein and oxidative stress, ER stress activates UPR via stress sensors and is targeted for degradation by p62/SQST1. Activated p62/SQST1 by TRAF2 and targets Keap1 and IKK, enabling nuclear localization of Nrf2, expressing antioxidant genes along with anti-inflammatory gene and NF-kB expressing inflammatory response respectively

Understanding the interplay between the AMPK pathway and the NF-κB pathway is crucial in neuroinflammation and neurodegeneration. The energy sensors AMPK and NF-κB are transcription factors that regulate proinflammatory responses. During starvation, AMPK promotes a catabolic process for ATP production by inhibiting anabolic processes that consume ATP. According to previous findings, upregulated AMPK has anti-inflammatory effects, activated AMPK promotes an anti-inflammatory response in microglia (Saito et al. 2019; Velagapudi et al. 2017). During oxidative stress, activated AMPK phosphorylates Nrf2 ser558 residue, causing Nrf2 nuclear aggregation and upregulation of antioxidative genes (Joo et al. 2016). AMPK promotes an anti-inflammatory response by preventing NF-kB nuclear localization, thereby diminishing proinflammatory cytokine expression (Xiang et al. 2019). In studies conducted by Giri et al. (2004), AMPK agonists blocked the inflammatory response of glial cells (Giri et al. 2004). Activated AMPK phosphorylates downstream signaling molecules such as FoxO and SIRT1, subsequently suppressing inflammatory gene expression and inhibiting inflammation (Giri et al. 2004; Xiang et al. 2019). Xiang et al. (2019) reported a reduction in the expression of IL-1β, a proinflammatory cytokine, and NF-κB inhibition in 8–9-week-old C57BL/6 mice upon AMPK activation, whereas inhibition reversed these effects (Xiang et al. 2019) (Fig. 5). Aberrant protein aggregation and oxidative ER stress activate UPR via sensors IRE1, PERK, and ATF6. At equilibrium BiP/GRF78 attached to sensors luminal domains, on stress stimulus, sensors dissociate from BiP/GRP78, activating UPR to halt protein synthesis and maintain protein homeostasis (Read and Schröder 2021). NF-kB activates through ER stress via UPR, primarily through the IRE1 pathway. Under stress, IKK activates through sensor IRE1 via TRAF2 recruitment. Activated IKK inhibits IkB by phosphorylating and leading to degradation, facilitating NF-kB dimers nuclear translocation. ER stress sensors IRE1 also activates the JNK pathway and promotes AP-1-mediated inflammation. Another ER stress sensor, PERK, activates NF-kB by inhibiting IkBα (Lin et al. 2019). Pro-inflammatory IL-1 activates adapter p62 via TRAF2, and activated p62 triggers IKK and phosphorylates IkB, leading to IkB degradation enabling NF-kB nuclear localization (J. Zhang et al. 2016). Impaired autophagy reduces p62/SQST1 autophagic degradation, causes cytoplasmic accumulation, and deregulates p62/SQST1/Nrf2/Keap1 system. Impaired Nrf2 disrupts redox homeostasis and deteriorates neurons (Armeli et al. 2024).

p62/Nrf2/KEAP1 plays a vital role in cellular response and protects against inflammation and oxidative stress. Upon stress stimuli, p62 sequestered and degrades Keap1 from the Nrf2 complex by ubiquitination process and facilitating Nrf2 nuclear translocation, where it interacts with antioxidant response elements and upregulates the expression of antioxidant enzymes such as glutathione s-transferase (GST), heme oxygenase-1 (HO-1) (Kong et al. 2021; Saito et al. 2019; Velagapudi et al. 2017; Wei et al. 2019). During ER stress, p62/ SQST1 facilitates ubiquitinated protein for autophagosomal degradation and simultaneously activates autophagy with Nrf2 (Jiang et al. 2015). Studies conducted in ICR mice (Nrf2^+/+^) and Nrf2-deficient mice (Nrf2^−/−^) concluded that AMPK influences the antioxidative pathway Nrf2. The activation of Nrf2 results in increased expression of antioxidative and anti-inflammatory genes in the inflammatory response and minimized oxidative stress (Mo et al. 2014). Another research focused on inflammation and oxidative stress induced by rotenone in BV2 cell lines, cells treated with resveratrol (50 μM & 100 μM) show reactive microglial attenuation compared with single resveratrol treatment. Nrf2 expression upregulated while downregulation of Keap1 and inhibited IL-6 and IL-1β in resveratrol treatment (Sun et al. 2021). Administration of 1 μmol curcumin on ICR mice back showed Nrf2 overexpression along with upregulated cytoprotective HO-1 level by cysteine modification of Keap1 (Shin et al. 2020). Bioflavonoids quercetin exerts anti-inflammatory and anti-oxidants, shows inhibition of PGE2, and downregulate COX-2, iNOS, and NF-kB expression in Wistar rats upon 25 mg/kg quercetin oral administration (Pany et al. 2014). Therapeutics development for antioxidants via targeting Nrf2 pathways is a promising approach in neurodegenerative therapy. Nrf2 is described as a cell guardian, protecting from oxidative stress. Conversely, aberrant and constitutive Nrf2 activation have adverse consequences, such as promoting tumorigenesis, allowing cancer cells to escape apoptosis through upregulated anti-apoptotic protein BCL2 and oncogenic genes Ras and Raf and facilitating resistance to chemotherapy by defective downstream signaling cascade (Hu et al. 2013; Sajadimajd and Khazaei 2018). Studies conducted on 3xTg-AD mice show elevated levels of PBMCs p(Ser40)Nrf2 and Nrf2 in the brain cortex substantiate oxidative defense and reduced antioxidant expression, leading to ER stress (Mota et al. 2015).

## Conclusion and future perspective

Cellular senescence and autophagy have complex and dynamic interactions since they involve immune cells in the brain. Autophagy is essential for preserving cellular homeostasis and reducing stress-related responses. Cellular senescence is a defense process that restricts the growth of damaged cells. The aging brain, the expressed dysregulated neuroimmune system, and defective autophagy mechanisms cause toxic proteins and damaged organelles to aggregate and cause cellular senescence, which exacerbates neuroinflammation and further causes neurological disorders.

Immune cell associations, including those of microglia and astrocytes, are crucial for maintaining brain function. The autophagy of microglia is crucial for controlling a balance between their neuroprotective and proinflammatory effects. Similarly, autophagy prevents astrocytes from aging and producing toxic chemicals, preserving their capacity to support neurons. Understanding these processes is essential for assessing brain aging and neurodegeneration.

Future studies should aim to elucidate the molecular mechanisms and the signaling networks that govern the crosstalk of autophagy and detection by immune cells of the brain with special focus on method development for imaging biomarkers in early neuroinflammation detection. Techniques such as single-cell transcriptomics and proteomics are next-generation methods and can determine cell-specific responses with relevance to cell type, thereby being vital for the context of the brain. This would also be important to provide accurate models of aging and neurodegenerative conditions that are real-life situations for the successful translation of basic research into effective therapeutic interventions.

Therapeutically, addressing the autophagy‒senescence pathway has tremendous promise. Senescent cells may be eliminated, and their cellular function may be restored with the use of autophagy-enhancing techniques such as genetic or pharmaceutical therapies. Senolytics, target senescent cells specifically, and senomorphics, which alter their secretory phenotype, may lessen the negative consequences of cellular decline in the brain. Neurodegeneration progression may slow down, or combining these strategies with currently available therapies may reverse it.

The development of novel treatments aimed at reducing neuroinflammation, encouraging neuronal regeneration, and maintaining cognitive function in aging and neurodegenerative diseases may be facilitated by a better understanding of the interaction between autophagy and senescence in immune cells in the aging brain. Novel approaches to addressing brain health issues in aging populations will be made possible by merging fundamental and translational research in this field.

## Data Availability

No datasets were generated or analysed during the current study.

## Abbreviations

NDDs: Neurodegenerative disorders
PD: Parkinson’s disease
AD: Alzheimer’s disease
CNS: Central nervous system
BBB: Blood–brain barrier
IGFBPs: Insulin-like growth factor-binding proteins
CSF: Colony-stimulating factor
PGE2: Prostaglandin E2
MMPs: Matrix metalloproteinases
NLRP3: NOD-like receptor pyrin domain containing 3
NFTs: Neurofibrillary tangles
IGFBPs: Insulin-like growth factor-binding proteins
CSF: Colony-stimulating factor
PGE2: Prostaglandin E2
